# Detection of sub-microscopic blood levels of *Plasmodium falciparum* using Tandem Oligonucleotide Repeat Cascade Amplification (TORCA) assay with an attomolar detection limit

**DOI:** 10.1038/s41598-019-39921-9

**Published:** 2019-02-27

**Authors:** Andrey L. Ghindilis, Olga Chesnokov, Billy Ngasala, Maria W. Smith, Kenneth Smith, Andreas Mårtensson, Andrew V. Oleinikov

**Affiliations:** 1 TORCATECH, LLC, 5210 104th Street SW, Mukilteo, WA 98275 USA; 2 Department of Biomedical Science, Charles E. Schmidt College of Medicine, Florida Atlantic University, 777 Glades Road, Boca Raton, FL 33428 USA; 3 Department of Parasitology and Medical Entomology, Muhimbili University of Health and Allied Sciences, Dar es Salaam, Tanzania; 4 Department of Women’s and Children’s Health, International Maternal and Child Health (IMCH), Uppsala University, Akademiska sjukhuset, 751 85 Uppsala, Sweden

## Abstract

Tandem Oligonucleotide Repeat Cascade Amplification (TORCA) based on signal rather than target amplification under isothermal conditions was developed for nucleic acid assays. The initial signal was generated by hybridization of single stranded DNA targets to immobilized recognition probes followed by hybrid cleavage with specific restriction endonuclease (REase), and release of trigger oligonucleotides (Tr1). The signal amplification chamber contained two bead types carrying single-stranded amplification probes and two amplification REases. The probes consisted of multiple tandem repeats of either Tr1 or another trigger Tr2, with the tandem-Tr1 anchored to the beads through the antisense Tr2 linker and vice versa. Addition of the recognition reaction solution and Tr1 hybridization to the anti-Tr1 linkers started cleavage and release of additional Tr1 and Tr2, resulting in exponential signal amplification. The cleavage cascade also released horseradish peroxidase (HRP) pre-attached to the amplification probes, and the resultant signal was measured colorimetrically. A TORCA assay was developed for detection of *Plasmodium falciparum* parasites in blood. It had the detection limit in the attomolar concentration range, successfully detecting sub-microscopic *P*. *falciparum* infections at less than 0.75 infected erythrocytes per microliter. Further TORCA optimization will likely produce the quantitative isothermal alternative to PCR at a fraction of its cost.

## Introduction

Sensitive and specific diagnostic methods using field-friendly approaches are extremely valuable for timely detection of infectious diseases. This is especially true for malaria, as successful elimination and eradication campaigns would benefit substantially from mass screening methods that can detect sub-microscopic levels of parasitemia. Presently, the most sensitive methods rely on nucleic acid amplification producing multiple copies of a target of interest for subsequent detection. This approach is used for the current gold standard, quantitative PCR (qPCR) that is sensitive enough to detect very low target amounts: dozens to hundreds of particular DNA molecules^1–3^. The main disadvantage of qPCR-based assays for field applications is the requirement for thermocycling and thus, the use of high cost, complex and bulky instrumentation. Frequent problems of qPCR include non-specific primer binding^1,2^, and high susceptibility to presence of various contaminants^3^. Currently, the main alternative to qPCR technology is the loop-mediated isothermal amplification (LAMP). It involves four to six custom-designed primers recognizing six distinct regions of a target DNA sequence and is based on a DNA polymerase enzyme with high strand displacement activity in addition to replication activity^4,5^. The LAMP technology is performed at constant temperature of 60–65 °C and has several advantages over qPCR in terms of costs and ease of use. However, it also has major limitations associated with the requirement to design and optimize 4–6 primers for each new DNA target. Thus, an assay platform is needed that would combine high sensitivity and specificity of detection with the isothermal mode, design simplicity, and low costs of components and equipment.

Previously we have described two related DNA assay platforms based on specific restriction endonuclease (REase) cleavage: (i) Direct Restriction Assay (DRA)^6^ and (ii) Restriction Cascade Exponential Amplification (RCEA)^7^. Our data showed that RCEA could achieve sensitivity similar to qPCR by using exponential amplification of the assay signal, rather than by generating additional copies of target DNA as in qPCR. The REase-driven enzymatic cascade implemented for the RCEA signal amplification resulted in DNA detection with attomolar sensitivity^7^. In addition, RCEA showed nearly absolute specificity due to the two independent biorecognition steps involved, it was insensitive to contamination, and the resultant assays provided for the desired isothermal mode, design simplicity, and low associated costs. The main RCEA drawback was the necessity to conjugate a REase enzyme with an oligonucleotide probe. Such conjugation is best achieved using SH- containing cysteines of the enzyme, however, the naturally occurring ones may be inaccessible by being buried inside the molecule, and/or their modification may destroy the catalytic activity. Therefore, the RCEA technology required the REase engineering for ligand attachment by identifying surface non-essential amino acid residues that could be mutated into cysteines^7^. Such approach could increase the assay costs and limit the REase selection for assay design. Thus, we have developed an approach alternative to RCEA that is also based on the REase-driven exponential signal amplification but does not require REase conjugation or modification. The novel method could be used with off-the-shelf commercial enzymes to achieve the desired low costs and design flexibility.

**Figure 1:**
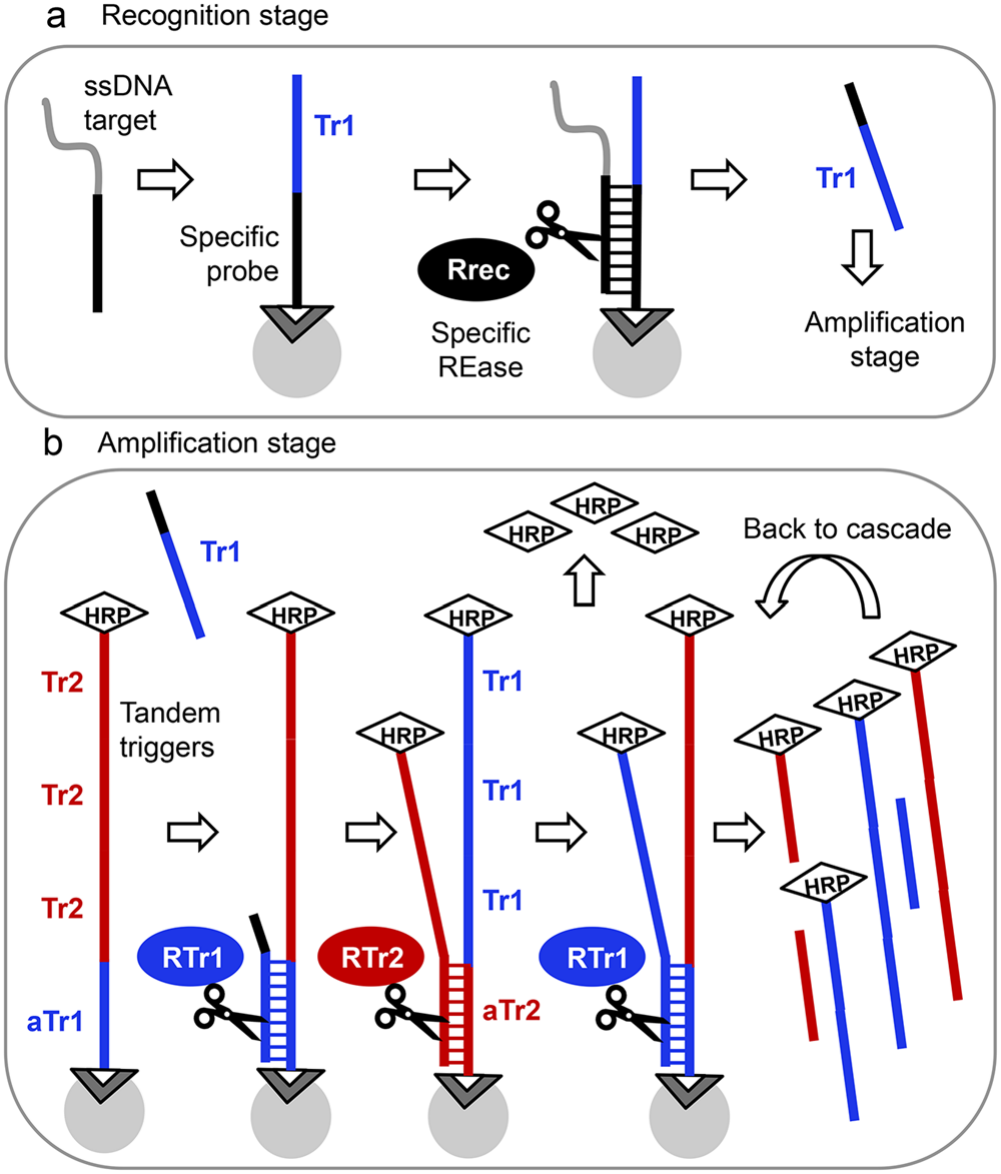
Tandem Oligonucleotide Repeat Cascade Amplification (TORCA). **(a)** The recognition stage schematic. An oligonucleotide probe specific for a target of interest is extended with a ‘trigger’ unit (Tr1) and attached to surface using biotin. A test sample containing the target of interest is added. The target in the test sample hybridizes to the probe, and the hybrid is specifically cleaved by specific recognition REase (Rrec) that is present in solution. The Tr1 unit is subsequently released into the reaction solution. (**b**) The amplification stage schematic. The reaction chamber contains two types of amplification probes. First contains a single unit complementary to the trigger sequence Tr1 (antisense Tr1, aTr1), and multiple identical units of a ‘trigger’ sequence Tr2. Second contains the multiple identical Tr1 units, and a single unit complementary to the Tr2 unit (antisense Tr2, aTr2). Both probe types are surface-attached and contain a molecular marker HRP on their solution-facing end. The reaction solution in the amplification chamber contains two common REase enzymes, ReTr1 and ReTr2, that recognize and cleave dsDNA hybrids of Tr1-aTr1 and Tr2-aTr2, respectively. When the recognition reaction solution is transferred to the amplification chamber, the free trigger Tr1 hybridizes to the aTr1 unit of the first probe leading to the probe cleavage by ReTr1 and release of Tr2 into the reaction solution. In turn, the released Tr2 hybridize to the aTr2 of the second probe type, causing cleavage with ReTr2 and further release of additional Tr1 units. Tis cascade of events also results in release of the HRP molecular marker that can be used for signal quantification.

The technology described in this report is based on Tandem Oligonucleotide Repeat Cascade Amplification (TORCA), a novel principle that is shown in Fig. 1. The TORCA assay consists of two stages: recognition (Fig. 1a) and amplification (Fig. 1b). A reaction chamber for the recognition stage contains a recognition probe that is attached to solid surface and, thus, unable to diffuse into the reaction solution. The recognition probe consists of two sequence units. First unit is assay-specific, it is complementary to the target of interest and contains a restriction site for a target-specific recognition REase enzyme. The second unit, a ‘trigger’ sequence (Tr1) (Fig. 1a) is common for various assays, and it is included to start the amplification cascade at the second stage of the TORCA assay (Fig. 1b).

Addition of ssDNA targets to the recognition chamber results in hybridization with the target-specific unit of the recognition probe that is followed by the enzymatic cleavage of the hybrid by the target-specific recognition REase (Fig. 1a). This cleavage releases the ‘trigger’ unit (Tr1) from the surface into the reaction solution. The reaction solution is then transferred into the amplification chamber for the second TORCA stage.

The amplification reaction chamber contains two types of amplification probes immobilized on solid surface (Fig. 1b). The first type contains a single unit complementary to the trigger sequence Tr1 (antisense Tr1, aTr1), and multiple identical units of a ‘trigger’ sequence Tr2. The second probe type contains the multiple identical Tr1 units, and a single unit complementary to the Tr2 unit (antisense Tr2, aTr2) (Fig. 1b). Both probe types are surface-attached and contain a molecular marker HRP on their solution-facing end. The reaction solution in the amplification chamber contains two common REase enzymes, ReTr1 and ReTr2, that recognize and cleave dsDNA hybrids of Tr1-aTr1 and Tr2-aTr2, respectively. Since the amplification probes are immobilized, physically separated, and single-stranded, no cleavage occurs in the chamber in the absence of free Tr1 and Tr2 units.

Addition of the reaction mixture from the recognition chamber containing some free Tr1 units starts the enzymatic cascade in the amplification chamber. Tr1 hybridizes with the first type of amplification probes, and the hybrid is cleaved by the ReTr1 enzyme, releasing multiple Tr2 units into the reaction solution. In turn, free Tr2 hybridize with the second type of amplification probes initiating cleavage by the ReTr2 enzyme and release of numerous additional Tr1 units (Fig. 1b). Since multiple units are released in each amplification cycle, the cascade amplifies the initial Tr1 signal exponentially. Each cleavage is accompanied by release of the HRP marker thus enabling signal quantification. In contrast to the recognition stage that is target-specific, the amplification stage design can be common for all targets and does not require modification for new analytes.

The goals of this study were (i) to develop and optimize the TORCA-based assays in order to reach the detection limit in the attomolar range, comparable to qPCR and RCEA; (ii) to demonstrate high assay specificity in complex mixtures containing both target and foreign DNA, (iii) to prove the assay feasibility for analytes of practical significance. One of such analytes is the malaria parasite *Plasmodium falciparum*. An important task for malaria elimination campaign is the development of field-deployable and highly sensitive detection methods. These methods are essential for early diagnosis, and for mass screening of malaria parasite carriers that should include small children, and thus, operate with small-volume samples <100 µL of blood. To further improve the detection sensitivity, we used malaria-specific RNA targets, since the amounts of RNA per cell can greatly exceed (up to several orders of magnitude) the amounts of DNA. The developed TORCA-based assays were successfully tested using simulated laboratory samples and blood of malaria-infected patients undergoing artemether-lumefantrine treatment in Bagamoyo, Tanzania.

## Methods

### Oligonucleotide probes for recognition and amplification stages

Table 1 provides a list of oligonucleotides purchased from Integrated DNA Technologies (Skokie, IL). Three 5′-end biotinylated oligonucleotides bio-polyC-ST-SP, bio-polyC-CL-SP, and bio-polyC-HN-SP were used as recognition stage probes to detect three different targets: AST and ACL, corresponding to fragments of A-type 18S rRNA, and AHN designed for a fragment of Translation Elongation Factor 2 gene of *P*. *falciparum*.

**Table 1:** Oligonucleotides used for the TORCA assay designs.

Name	Usage	Sequence	5′ mod	REase	Length
Thiol polyC	HRP tag	CCCCCCTTTCCCCCCCCC	Thiol	No	18
Bio-polyA-ARV(GT)	Primer	AAAAAAAAAAAAGATGTTAG**GATATC**TTGTATAGGT	Biotin	EcoRV	36
Bio-polyA-ASP(CTAT)	Primer	AAAAAAAAAAAATGTTGAG**AATATT**ACTAGACCTAT	Biotin	SspI	36
polyC	Primer	CCCCCCTTTCCCCC	No	No	14
Bio-polyC-ST-SP	Recognition probe	CCCCCCGTATTTGTT**AGGCCT**TATAAGAGTCTAGT**AATATT**CTCAACA	Biotin	StuI, SspI	48
Bio-polyC-CL-SP	Recognition probe	CCCCCCTCAAAGA**ATCGAT**ATTTTATTGGTCTAGT**AATATT**CTCAACA	Biotin	ClaI, SspI	48
Bio-polyC-HN-SP	Recognition probe	CCCCCCTTCATGA**AAGCTT**ATCCATTAGGTCTAGT**AATATT**CTCAACA	Biotin	HindIII, SspI	48
AST	Target	TCTTATA**AGGCCT**AACAAATAC	No	StuI	22
ACL	Target	CAATAA AAT**ATCGAT**TCTTTGA	No	ClaI	22
AHN	Target	CTAATGGAT**AAGCTT**TCATGAA	No	HindIII	22
ARV-7SP-polyG	Template	GATGTTAG**GATATC**TTGTATAG7x[GTCTAGT**AATATT**CTCAACA]GGGGGGGGGAAAGGGGGG	No	EcoRV, SspI	180
ASP-7RV-polyG	Template	TGTTGAG**AATATT**ACTAGAC7x[CTATACAA**GATATC**CTAACATC]GGGGGGGGGAAAGGGGGG	No	SspI, EcoRV	192
SP	Trigger 1	GTCTAGT**AATATT**CTCAACA	No	SspI	20
RV	Trigger 2	CTATACAA**GATATC**CTAACATC	No	EcoRV	22

Bold lettering in the oligonucleotide sequences indicates the REase recognition sites, with the corresponding enzymes listed in the ‘REase’ column.

**Figure 2:**
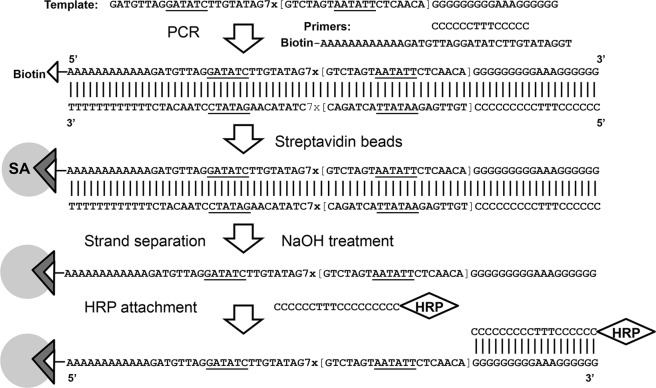
Amplification probe preparation and attachment to magnetic bead surface illustrated using AR(7SP)-polyG (Table 1).

Large scale generation of the biotinylated amplification probes was performed using PCR (Fig. 2) with template oligonucleotides ARV-7SP-polyG or ASP-7RV-polyG and primers bio-polyA-ARV(GT) or bio-polyA-ASP(CTAT), respectively. Biotin-free polyC oligonucleotide was also added to both reactions. Phusion High-Fidelity PCR Kit (New England Biolabs, Ipswich, MA) was used with the templates added to 50 µL reactions at 1 nM concentrations. PCR conditions included 40 cycles of denaturing at 95 °C for 10 sec, annealing at 58 °C for 20 sec, and extension at 72 °C for 20 sec. The PCR product was then purified by using Invitrogen ChargeSwitch®-Pro PCR Cleaning Kit (Thermo Fisher Scientific Inc., Rockford, IL), and applied to Micro Bio-Spin columns with Bio-Gel P-6 (P-6 column) (Bio-Rad, Hercules, CA) pre-equilibrated with PBS. The final volume of the purified double-stranded amplification probe was adjusted to the initial PCR volume of 50 µL by adding PBS.

### HRP conjugate preparation

To attach the HRP marker to the amplification probes, HRP was conjugated to the thiolated oligonucleotide polyC (thiol-polyC) as previously described^6^. The conjugation reaction was done with excess of HRP over to the oligonucleotide, thus, the final conjugate concentration was equivalent to the initial polyC concentration. The resultant HRP-linked tag was used to label the 3′ ends of amplification probes by hybridizing to polyG.

### Attachment of amplification probes to magnetic beads

A schematic of the entire preparation procedure is shown in Fig. 2 for the amplification probe ARV(7SP)-polyG and polyC-HRP. Hydrophilic Streptavidin Magnetic Beads suspension (400 µL of 4 mg/mL stock, New England Biolabs) was settled and washed 2 times with 400 µL PBS. Then the beads were resuspended in 400 µL of 200 mM sodium hydroxide for 5 min to remove weakly bound streptavidin^8^. The beads were then washed twice with 10X PBS for pH neutralization and 3 times with 1X PBS. The pre-washed beads were used for biotin-streptavidin attachment by mixing with 400 µL of the purified double-stranded amplification probes (diluted 1:1 with 1X PBS). The resultant suspension was incubated at room temperature for 5 hours in a Labquake Shaker Rotisserie (Thermo Fisher Scientific) at 8 rpm. After incubation, the beads were washed 5 times with PBS, and then treated with 400 µL of 200 mM sodium hydroxide for 5 min to denature the attached double-stranded probes. The beads were then washed twice with 400 µL10X PBS and 3 times with 1X PBS to remove the non-biotinylated strand. The resultant beads carried the single-stranded amplification probes linked to surface through biotin-streptavidin interactions.

The bead-immobilized single-stranded amplification probes carried polyG on the solution end. It was used to attach the molecular marker by mixing the bead suspension with 800 µL of 1X PBS containing 150 nM HRP-polyC conjugate and 0.5 mg/mL of BSA. Following overnight incubation at room temperature, the beads were washed 10 times with 800 µL of 1X PBS and stored at 4 °C until use. The final working concentration of the beads in PBS was 2 mg/mL. Depending on the attached probe, the prepared beads were designated either ASP-7RV-polyG::polyC-HRP or ARV(7SP)-polyG:: polyC-HRP. The probes were single-stranded with the exception of the polyG::polyC hybrid at the 3′ ends. The beads were stored at 4 °C until use.

### Suspension testing of beads coupled to amplification probes

Five µL of bead preparations ASP-7RV-polyG::polyC-HRP and ARV-7SP-polyG::polyC-HRP (at 2 mg/mL concentrations) were placed into individual 0.5 mL Eppendorf tubes, and washed twice with 10 µL of CutSmart buffer (New England Biolabs). The first preparation, ASP-7RV-polyG::polyC-HRP, was used to add 5 µL of the target solution: either CutSmart buffer supplemented at various concentrations with the SP trigger oligonucleotide (Table 1), or a negative control of the same buffer with no targets added.

The second preparation, ARV-7SP-polyG::polyC-HRP, was used to add two REases, EcoRV and SspI, at 0.8 U/µL each, in 5 µL of CutSmart buffer. Finally, the two bead preparations were mixed together to start the REase-driven amplification cascade, and incubated at 37 °C in a rotisserie for a given time. When the SP triggers were present, they hybridized to the immobilized probes. After incubation, the beads were settled, and 8 µL of the supernatant containing cleaved oligo-HRP conjugate were collected for signal measurements. The supernatants were placed into microplate wells and mixed with 80 µL of BioFX TMB One Component HRP Microwell Substrate (SurModics, Eden Prairie, MN) to start the HRP colored reaction. The signal forming after 10–12 min incubation at room temperature was measured colorimetrically at the wavelength of 655 nM (OD_655_) using a Bio-Rad iMark Microplate Reader. All testing was performed in triplicate.

### Barrier-separated testing of beads coupled to amplification probes

To create physical barriers between the beads attached to different amplification probes, a 5 mm (in diameter) disk made of glass fiber filter grade A (I.W. Tremont Co., Inc, Hawtrone, NJ) was placed into a microplate well. Next, 10 µL of ARV-7SP-polyG::polyC-HRP modified bead suspension (2 mg/mL in CutSmart buffer) were deposited onto the filter. The suspension was overlaid with another filter, and finally, 10 µL of ASP-7RV-polyG::polyC-HRP modified beads (2 mg/mL in CutSmart buffer) were placed on the top filter. Magnetic field was then applied for 10 seconds to the bottom of the microplate to fix the beads onto the filters.

To start the signal amplification cascade, 20 µL of CutSmart buffer supplemented with a particular concentration of the SP trigger oligonucleotide, and 20 µL of CutSmart buffer containing two REases, EcoRV and SspI (0.8 U/µL each) were added to the top of the second filter. The resultant microplate was incubated for various time intervals at 37 °C with shaking (90 rpm). After incubation, 20 µL of 1X PBS were added to the well, mixed with the reaction solution, and 50 µL of the mixture containing beads were transferred into a new well. The residual beads were settled, and 35 µL of the supernatant were used for HRP signal measurement by mixing with 150 µL of BioFX TMB One Component HRP Microwell Substrate. The blue color HRP-generated signal was measured colorimetrically at the wavelength of 655 nM (OD_655_).

### Attachment of the recognition probe to magnetic beads

Hydrophilic Streptavidin Magnetic Bead suspension (200 µL of 4 mg/mL stock suspension, New England Biolabs) was settled and washed 2 times with 200 µL 1X PBS. Then the beads were resuspended in 200 µL of 200 mM sodium hydroxide for 5 min to remove weakly bound streptavidin^8^. The beads were then washed twice with 10X PBS and 3 times with 1X PBS. Finally, the pre-washed beads were incubated with 200 µL of 1 µM solution of a recognition probe oligonucleotide (bio-polyC-ST-SP, bio-polyC-CL-SP, and bio-polyC-HN-SP, Table 1) or mixture of all three recognition probes (1 µM each) in 1X PBS. Each immobilized probe contained the gene-specific unit (ST, CL, or HN) and the common SP trigger unit. The resultant suspension was incubated at room temperature for 5 hours in a Labquake Shaker Rotisserie (Thermo Fisher Scientific) at 8 rpm. After incubation the beads were washed 10 times with 1X PBS and stored at 4 °C until use.

### Integration of recognition and amplification tests

The recognition beads were transferred into 1x SSPE buffer to prepare 2 mg/µL suspension. Bead suspension (10 µL) was then separated in the magnetic stand and supplemented with 40 µL of SSPE buffer containing the corresponding target (AST, ACT, AHN), complementary to each gene-specific unit (Table 1), and added at a particular concentration. For samples containing cDNA prepared from *P*. *falciparum* infected blood, the salt concentration in the preparation was adjusted to 1x SSPE by adding the appropriate amount of 20x SSPE concentrate. The reaction suspension was incubated for 40 min at 37 °C in a rotisserie to achieve target to probe hybridization. The beads were then washed twice with 1x SSPE buffer and 3 times with CutSmart buffer. Then 20 µL solution of 0.4 U/µL of corresponding REase or mixture of two REases (Table 1) was added. The beads were resuspended and incubated for 40 min at 37 °C in a rotisserie to facilitate the enzymatic cleavage of probe-target hybrids and to release the SP units into the reaction solution. After completion, the supernatant containing the released SP was used for testing with the amplification beads as described above.

### Preparation of nucleic acids from *P*. *falciparum* samples

The laboratory parasite strain NF54 was grown to 2.5% parasitemia in 2% hematocrit as previously described^9^. This resulted in 1.7 × 10^6^ IE/mL of culture by cytometer cell counting. Serial 10-fold dilutions of this culture were prepared using purified red blood cells in RPMI (at 50% hematocrit) to imitate human blood cultures with various parasitemia. Thus, the most concentrated sample #1 had 1.7 × 10^5^ IE/mL and the least concentrated #5 had 17 IE/ml. To preserve RNA, 2.5 volumes of RNAgard Blood Reagent (Biomatrica, San Diego, CA) were added to each sample, and the samples were stored either at 4 °C for immediate use (up to one month) or frozen at −30 °C for long term storage according to the manufacturer’s instructions.

Total RNA was isolated from 350 µL of the culture dilution/RNAgard mix (corresponding to 100 µL of infected blood) using E.Z.N.A. Blood RNA Kit (OMEGA Bio-tek, Norcross, GA). The protocol was slightly modified for preparation of *P*. *falciparum* RNA: 1/3 of the sample volume (100 µL) of water was added, mixed by pipetting, and then the mixture was centrifuged for 10 min at 3,000 rpm in a tabletop centrifuge. Supernatant was discarded, and 500 µL of ERL-buffer was added to the pellet. The suspension was vortexed and incubated on ice for 5–20 min with repetitive vortexing during incubation. Then 500 µL of NTL lysis buffer with 2-βME (20 µL/1 mL) was added and mixed by pipetting. Samples were transferred into homogenizer mini columns, and centrifuged for 2 min at 10,000 × *g*. The flow-through was collected, mixed with an equal volume of 70% ethanol, and passed through HiBand RNA mini columns. After centrifugation for 30 sec at 10,000 × g, the flow-through was discarded, and the columns were washed with 500 µL of RWF wash buffer using 30 sec centrifugation. Then the columns were washed with 700 µL of RNA wash buffer II twice. RNA was eluted with 100 µL of water using 5 min incubation at room temperature and centrifugation for 2 min at 10000 × *g*. The resultant purified RNA samples from the parasite serial dilutions were stored at −80 °C. Each RNA sample was used to apply 1 µL for the reverse transcription-PCR titration curve (section “Reverse Transcription-PCR quantification of *P*. *falciparum* samples” below). For TORCA analyses, the purified RNA samples were converted into cDNA using the reverse transcriptase reagents from the Superscript III kit (Invitrogen, Thermo Fisher Scientific). Each 100 µL reaction included 50 µL of 2x buffer containing dNTPs, the enzyme mixture (SuperScript® III Reverse Transcriptase and Platinum® Taq DNA polymerase), random hexamers (Invitrogen, Thermo Fisher Scientific), RNasin (Promega, Madison, WI), and 50 µL of template RNA. Reactions were incubated for 1 h at 50 °C. Then the reactions were treated with 1 µL of Ribonuclease H (Applied Biosystems, Foster City, CA) for 20 min at 37 °C. Finally, 10 µL of the cDNA preparations were used for TORCA assays.

### Human sample collection and usage

Venous blood samples (3 ml) were collected during artemether-lumefantrine treatment study in Bagamoyo, Tanzania from patients aged between 6 months and 10 years, as a part of an ongoing and approved clinical study (Tanzania Evaluation and Surveillance of ACT (TES) efficacy supported by the National Malaria Control Program) conducted in May-July 2018. Samples were collected at day “0” and days 1, 2, 3, 7, 14, 21, and 28 after the beginning of treatment. After collection, samples were mixed with 2.5 volumes of RNAgard Blood Reagent and stored at −80 °C before shipping to the Florida Atlantic University in frozen blocks of blue ice. After arrival samples were stored in accordance with the RNAgard manufacturer’s instructions at −80 °C and thawed on ice before use. Convalescence samples from a patient #3 with the initial parasite concentration of 1.4 × 10^6^ IE/mL (as evaluated by microscopy on day “0”) were selected for parasite detection at days 1, 7, and 14 of treatment by PCR and TORCA. Samples corresponding to 100 µL of blood were used to isolate total RNA and convert it to cDNA as described above. Alternatively, 10 µL of isolated RNA was used for reverse transcription-PCR (as described below).

### Reverse transcription-PCR quantification of *P*. *falciparum* samples

Reverse transcription-PCR was performed using Superscript III with platinum *Taq* One step Kit (Invitrogen, Thermo Fisher Scientific). A typical 25 µL reaction contained 12.5 µL of 2x buffer, 1 µL of mixture of forward and reverse primers (10 µM each), 1 µL of the enzyme mix, 1 µL of template RNA and 9.5 µL of RNase free water. The reaction primers PL1473F18 (5′-TAACGAACGAGATCTTAA-3′, Tm = 44.6 °C) and PL1679R18 (5′-GTTCCTCTAAGAAGCTTT-3′, Tm = 46.1 °C) previously described elswhere^10^ have been designed to amplify a 214 bp fragment of *P*. *falciparum* 18S rRNA. The following PCR conditions were used: (1) **cDNA synthesis**, 1 cycle: 50 °C for 30 min; (2) **denaturation**, 1 cycle, 94 °C for 2 min; (3) **PCR amplification**, 30 cycles, 94 °C for 15 sec, 48 °C for 30 sec, 68 °C for 1 min; (4) **final extension**, 1 cycle, 68 °C for 10 min. The samples were analyzed using 2% TAE agarose gels with ethidium bromide staining. Gel image analysis was done using the UVP EpiChemi II Darkroom (UVP Laboratory Products), and the DNA bands were quantified with the Zen 2012 Blue software. The reverse transcription-PCR approach was tested with the titration series of five simulated infected blood samples with 10-fold differences in parasitemia (described above). The reaction conditions were optimized to achieve linear graphs of the dependence between the assay fluorescent signals and the parasite concentrations when plotted using the semi-log scale (Supplementary Fig. 1). The graphs then served as standard curves for the PCR quantification of parasitemia in unknown field samples.

### Ethical approval and informed consent

Collections of blood samples were carried out in accordance with the relevant guidelines and regulations. Informed consent was obtained from all children’s legal guardians and assent from all participants. The use of human blood samples and all methods used in work with human blood were approved by Tanzanian Muhimbili University of Health and Allied Sciences, the National Institute for Medical Research, and Florida Atlantic University Institutional Review Board.

## Results

### Signal amplification system design

Two oligonucleotide units, SP and RV (Table 1), were designed to contain the recognition sites for REases SspI and EcoRV, respectively. The units served as the amplification triggers Tr1 and Tr2 shown in Fig. 1b. The amplification probes were then designed to carry seven repeats of either SP (7SP) or RV (7RV) units. In addition, the 7SP probe carried a single unit complementary to RV (aRV), and vice versa, the 7RV probe had a single unit complementary to SP. The probes were produced using PCR (Fig. 2) with the goals to (i) label the 5′end with biotin for bead attachment, and (ii) introduce a 12-adenosine spacer between the 5′end biotin and the probe. The spacer was used to extend the distance between the restriction sites and the solid surface and thus, to improve efficiency of REase cleavage of surface-attached DNA.

Streptavidin coated magnetic beads were used as a solid carrier for the amplification probe attachment through biotin-streptavidin interactions. The immobilized probes were double-stranded (dsDNA), thus, the standard alkaline treatment with 200 mM NaOH was used to remove the complementary non-biotinylated strand^8^. This relatively mild treatment was likely insufficient to ensure complete probe conversion to the single-stranded (ssDNA) state. However, the remaining immobilized dsDNA probes were subsequently cleaved by the amplification REases added to the reaction solution. Under the applied isothermal conditions, the cleavage products consisting of dsDNA could not hybridize to the immobilized ssDNA probes, and thus could not initiate the signal amplification cascade in the absence of ssDNA triggers.

The 3′end of the amplification probes was designed to contain a polyG stretch for attachment of the HRP molecular marker. HRP was chemically conjugated to polyC oligonucleotides (Table 1), and the following polyC to polyG hybridization resulted in the HRP attachment to the amplification probes.

### Amplification system characterization in the absence of triggers

Two types of bead suspensions with the attached probes, ARV-7SP-polyG::polyC-HRP or ASP-7RV-polyG::polyC-HRP, were used to design the amplification system and to analyze background signals in the absence of assay targets. The first reaction chamber design contained 1:1 mixture of the two suspensions (‘bead mixture’). The second design used glass filter barriers for physical separation of the two types of beads (‘barrier-separated’) while allowing for free movement of the reaction solution among compartments. For both designs, the reaction solution contained the corresponding specific amplification REases (one or both), but no oligonucleotide trigger (Tr1) added.

**Figure 3:**
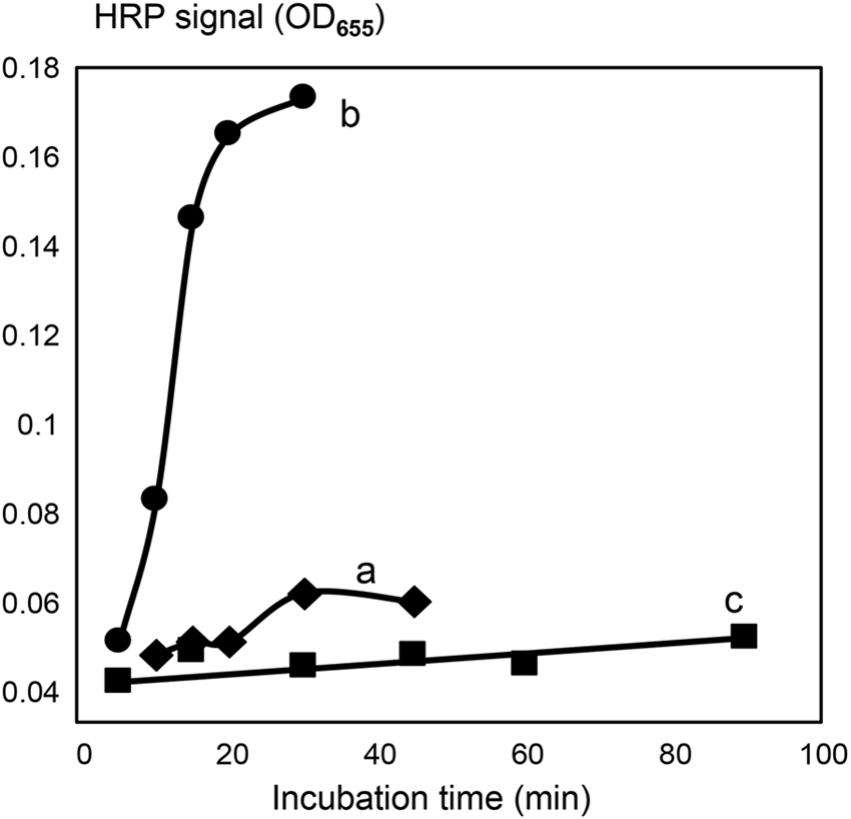
The dependence of assay signal on incubation time measured for the trigger-free reaction solutions. Two types of amplification chambers were used, the mixed bead and the barrier-separated designs. (**a**) The mixed bead design with a single REase, Rtr1 (SspI); (**b**) the mixed bead design with both REases: RtR1 and Rtr2 (SspI and EcoRV); (**c**) the barrier-separated design with both REases: RtR1 and Rtr2 (SspI and EcoRV). The X-axis shows the incubation time (min), and the Y-axis shows the background-subtracted and normalized HRP signal values. The background was calculated as the mean signal generated for the triplicate no-trigger added negative controls. For normalization and comparison of the sample series, the HRP signal values are expressed as the percentages of the maximum background-subtracted OD_655_ corresponding to each series. Error bars show standard deviations.

The bead mixture design provided for free interactions between the two types of beads, thus the ssDNA probes immobilized on one bead type could hybridize to the complementary probes immobilized on the other bead type. For example, the SP unit of the ARV-7SP-polyG::polyC-HRP probe could hybridize to the ASP unit of the ASP-7RV-polyG::polyC-HRP probe. Such hybridization resulted in the enzymatic cleavage of the probes by SspI REase, generating HRP signal. Figure 3 shows the dependence of the HRP-generated signal on the incubation time for the bead mixtures that contained either single SspI REase (A), or both EcoRV and SspI REases (B). Our data showed that the probe interaction in the bead mixture design could trigger a cascade of amplification reactions in the absence of triggers. In the single REase system (A), the observed HRP signal increase was slow, getting pronounced after 30 min incubation. In the two REase system (B), a sharp signal increase was observed promptly and then it continued to increase in the direct correlation with the incubation time.

In contrast, the ‘barrier-separated’ design prevented the direct contact of the two different bead types and probe-to-probe hybridization. Figure 3c shows the dependence of the HRP-generated signal on the incubation time for the barrier separated beads. These reactions contained both EcoRV and SspI REases, but no oligonucleotide triggers. Unlike the bead mixture, the barrier-separated bead design demonstrated almost no HRP signal increase in the absence of targets (Fig. 3c) even at the long incubation time of 90 min.

### Amplification system characterization in the presence of triggers

Experiments with addition of ssDNA trigger oligonucleotides at different concentrations showed that each developed amplification chamber design had specific advantages. The bead mixture design provided for overall simplicity and quick assay time due to the absence of diffusion limitations, however, it showed the significant background signal in the absence of targets (described above). Nevertheless, the addition of SP trigger increased the signal substantially and quickly, as compared to the background of negative controls with no trigger added.

**Figure 4:**
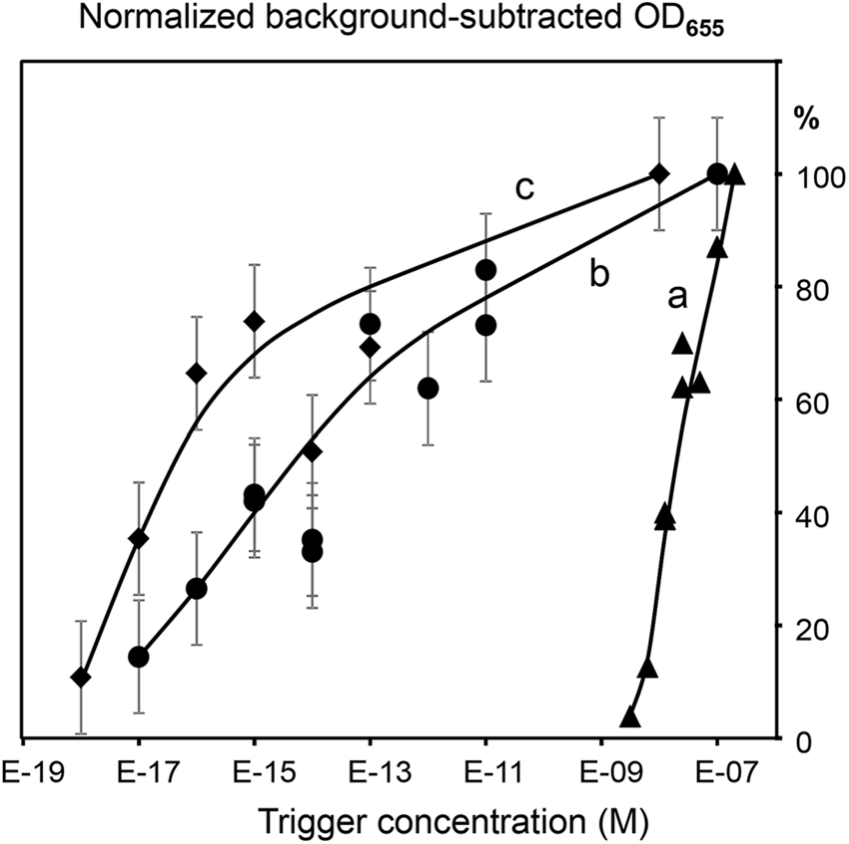
The dependence of the HRP-generated signal on the concentration of amplification trigger (SP) added to the single REase (**a**) or the two REase EcoRV and SspI (**b**) systems. The curves a and b are generated for mixtures of the two bead types, one modified with ASP-7RV-polyG-HRP and the other with ARV-7SP-polyG-HRP probes. The curve c was obtained for the same two bead types separated by a filter barrier. The X-axis shows the target concentrations (M), and the Y-axis shows the background-subtracted and normalized HRP signal values. The background was calculated as the mean signal generated for the triplicate no-trigger added negative controls. For normalization and comparison of the sample series, the HRP signal values are expressed as the percentages of the maximum background-subtracted OD_655_ corresponding to each series. Error bars show standard deviations.

Figure 4 shows the background-subtracted signal increases observed at short incubation times, i.e. 15 min after the SP trigger addition, in both single REase (a), or two REase (EcoRV and SspI) (b) systems of bead mixture designs. As expected, the single REase system (Fig. 4, curve a) had the lower detection limit in the nanomolar concentration range that was previously observed for such designs^6^. In contrast, the two REase system (Fig. 4, curve b) showed high sensitivity, and had the detection limit in the 10–100 attomolar range, similar to RCEA^7^ and comparable to qPCR. Thus, the TORCA exponential amplification provided for the 8 orders of magnitude improvement of the lower detection limit over the no-amplification system. The bead mixture design allowed to achieve high sensitivity using short incubation times. However, the drawback of this design was quick signal saturation and the necessity to precisely optimize the incubation time for each new prepared amplification bead batch. In the current study the batch to batch variation resulted in the optimum assay times varying from 13 to 16 min.

The barrier-separated system did not have high backgrounds and did not require optimization of the incubation time since the signal did not reach saturation even during prolonged incubations with the trigger oligonucleotides. Figure 4 curve c shows the results of the SP trigger addition to the barrier-separated beads containing two REases after 80 min incubation time. The lower detection limit was in the 10 attomolar range. However, the long incubation times required by this design indicate serious diffusion limitations that need to be mitigated by re-engineering of the reaction chamber. Thus, for the current proof-of-principle purpose we selected the mixture-based amplification design, and experimentally evaluated the optimum incubation time for each bead batch.

### TORCA-based detection of *P*. *falciparum* infections: assay targets design and testing of specific probes in model systems

The first assay designed using the developed TORCA system was focused on the malaria parasite *P*. *falciparum*. Our first step was selection of total RNA as nucleic acid targets for the assay development. By general estimates, ribosomal RNA (rRNA) is present at high copy numbers >10^6^ molecules/eukaryotic cell, and it is frequently used for qPCR-based detection of *P*. *falciparum* with high sensitivity^11^. Other potential RNA targets for qPCR are usually designed from highly expressed genes, like those encoding ribosomal proteins or translation factors known to be represented by >100 mRNA molecules/cell^12^.

Therefore, *P*. *falciparum*-specific recognition probes were designed using three gene fragments: two representing the A-type 18S rRNA species known to be expressed in the asexual form of parasite (most numerous in blood)^10^, and third representing the A-type 18S rRNA Translation Elongation Factor 2 (EEF2) mRNA. The 22-bp probes were selected to contain unique REase cleavage sites StuI, ClaI, and HindIII, with the respective names of ST and CL for the two targeting A-type 18S rRNA, and HN for the one targeting EEF2 (Table 1). The probes were designed in the sense orientation to enable detection of cDNA that is anti-sense. In addition to the gene-specific parts, each recognition probe contained the common oligonucleotide unit SP (for triggering of the amplification cascade) that was placed at the 3′ end facing the reaction solution after probe immobilization. The resultant recognition probes were designated Bio-polyC-ST-SP, Bio-polyC-CL-SP, and Bio-polyC-HN-SP (Table 1). Each probe type was individually immobilized on beads as described above.

**Figure 5:**
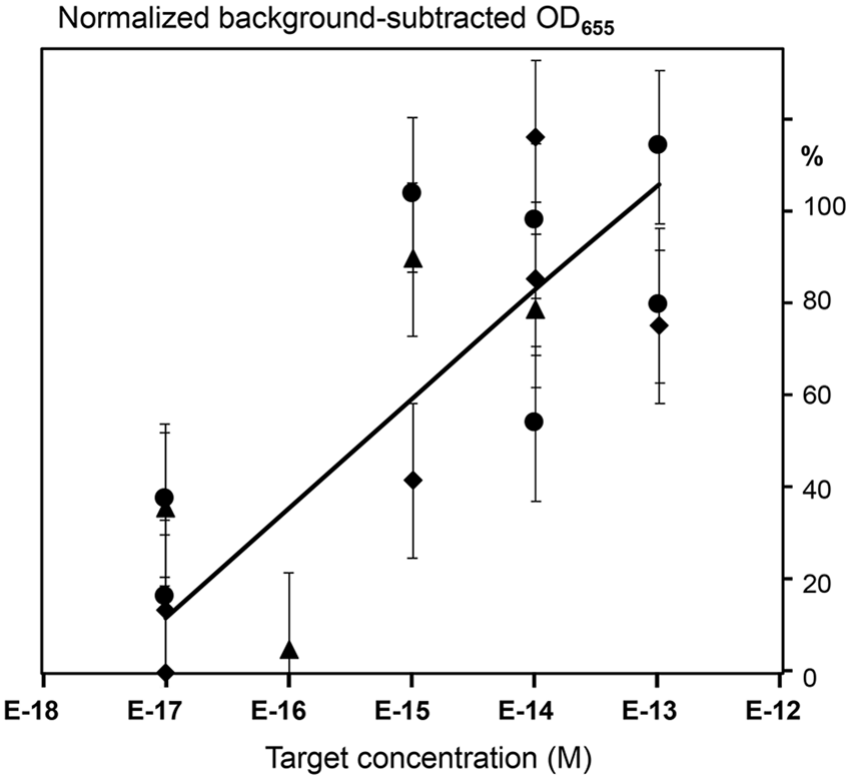
The dependence of the HRP-generated signal on the concentrations of targets AST (circles), ACL (triangles), and AHN (diamonds) that were added to the recognition stage. The X-axis shows the target concentrations (M), and the Y-axis shows the background-subtracted and normalized HRP signal values. The background was calculated as the mean signal generated for the triplicate no-trigger added negative controls. For normalization and comparison of the sample series, the HRP signal values are expressed as the percentages of the background-subtracted signal obtained at the highest concentration (10 nM) corresponding to each series. Error bars show standard deviations.

The three recognition bead suspensions were supplemented with the corresponding REase and then tested individually using the complementary oligonucleotides (AST, ACL, or AHN, Table 1) to simulate *P*. *falciparum* targets. After target hybridization to the recognition probes, the reaction solution containing free trigger SP units (released by the REase cleavage) was transferred to the bead mixture amplification chamber described above. The results are shown in Fig. 5 as the dependence of amplification signal on the concentration of oligonucleotide targets added at the recognition stage. The signals were background-subtracted using the negative controls with no targets added. All three oligonucleotide targets were successfully detected with very similar calibration curves showing the lower detection limit in the 10–100 attomolar range. Our results showed that the system response was proportional to the target concentration at the recognition stage, and that the different recognition probes provided for the similar detection efficiencies at the amplification stage.

Our previous data^7^ showed that the REase-based assays were insensitive to excess of foreign DNA, since this DNA did not hybridize with recognition probes and thus, was removed during washing steps. To characterize the TORCA assay for sensitivity to foreign DNA contamination, we added a commercial preparation of human genomic DNA at 1.4 pg/µL concentration to a sample containing 1 fM mixture of the target oligonucleotides AST, ACL, and AHN. Performance of this mixture was compared to the control sample with no foreign DNA added. Both samples showed very similar results (data not shown), indicating the assay tolerance of non-complementary DNA contamination.

### TORCA-based detection of *P*. *falciparum* in simulated samples and infected patient blood

Malaria detection approaches must be capable of testing small blood volumes <100 µL for mass screening of infected children. Thus, simulated blood infection samples were prepared at 100 µL volumes using purified red blood cells and *P*. *falciparum* NF54 parasite line-infected erythrocytes. The dilution series were prepared in a wide range of concentrations and used for total RNA isolation and reverse transcription into cDNA to apply to the developed TORCA assays.

To increase the assay sensitivity, the TORCA recognition chambers were designed to contain bead mixtures carrying all three developed recognition probes. Background signals were calculated based on the triplicate negative controls with no targets added. Then the average background values were subtracted from the test data values obtained with the cDNA preparations. In addition to the negative controls, each test series was supplemented with triplicate positive controls that contained an equimolar mixture of 10 nM concentrations of the target oligonucleotides AST, ACL, and AHN instead of cDNA. The mean positive control signal was used as the experiment-specific 100% reference, and the test dilution series data were normalized by expressing as the percentages of the corresponding reference.

**Figure 6:**
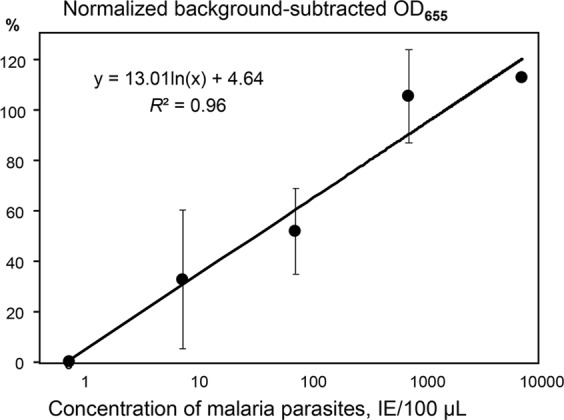
The dependence of the HRP-generated signal on the concentration of malaria parasites in the infected cells. The X-axis shows the concentrations (IE/100 µL), and the Y-axis shows the background-subtracted and normalized HRP signal values. The background was calculated as the mean signal generated for the triplicate no-trigger added negative controls. For normalization and comparison of the sample series, the HRP signal values are expressed as the percentages of the background-subtracted signal obtained for the positive control containing an equimolar mixture of AST, ACL, and AHN oligonucleotides at 10 nM concentrations. Error bars show standard deviations.

Figure 6 shows the dependence of the HRP generated signal on the parasite concentration in a test dilution series prepared from the simulated blood infections as described above. The resultant TORCA titration curve is linear on the semi-log graph, and it is similar to the titration curve obtained for the same samples using reverse transcription-PCR (Supplementary Fig. 1). Our data showed that the TORCA assay could detect malaria parasites at concentrations as low as 7.5 IE per 100 µL of simulated blood infection samples. This lower detection limit is more than an order of magnitude better than the target sensitivity of <200 IE/100 µL that is suggested by the World Health Organization (WHO) as the threshold for acceptance of a detection method to use in malaria elimination campaigns^13^.

**Figure 7:**
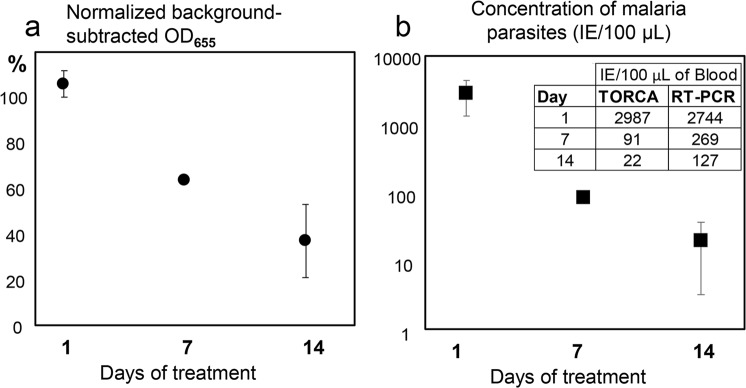
The dependence of the TORCA signal (**a**) and the calculated infected erythrocyte concentration (**b**) on the time of patient drug treatment (X-axis, days). (**a**) The Y-axis shows the background-subtracted and normalized HRP signal values. The background was calculated as the mean signal generated for the triplicate no-trigger added negative controls. For normalization and comparison of the sample series, the HRP signal values are expressed as the percentages of the background-subtracted signal obtained for the positive control containing an equimolar mixture of AST, ACL, and AHN oligonucleotides at 10 nM concentrations. (**b**) The Y-axis shows the IE concentrations calculated using the standard curve shown in Fig. 6. The inset shows mean data for IE concentrations measured using TORCA and reverse transcription-PCR methods and calculated according to corresponding calibration curves (Fig. 6 and Supplementary Figure 1, respectively). Error bars in both graphs show standard deviations.

As a proof-of-principle to further demonstrate that the TORCA assays could be used for quantitative analyses of field samples, we tested blood of an infected patient, who was treated with artemether-lumefantrin drugs. The initial sample at the beginning of treatment (day “0”) was characterized for parasitemia by microscopy analysis. We tested the convalescent samples after 1, 7, and 14 days of treatment by using the developed TORCA and PCR assays in parallel. The calibration curves obtained with the test dilution series of simulated *P*. *falciparum* infections (Fig. 6) were used to calculate the parasite concentrations in the patient blood. Figure 7a shows the TORCA signal dependence on the length of treatment, indicating that the amounts of parasite RNA decreased significantly in the patient blood with time. Figure 7b presents these data as the number of parasites per 100 µL of blood for 1, 7, and 14 days of treatment. The same patient samples were also quantified using PCR and the calibration curve shown in Supplementary Fig. 1. The inset table in Fig. 7b shows the direct comparison of parasite concentrations in the patient blood obtained with the two detection techniques. The data were consistent between the two approaches, indicating the gradual decrease in the parasite concentrations during treatment. Taken together, our data provide the clear proof-of-principle that the human malaria infection could be detected using TORCA assays at the parasitemia levels close to or below the WHO-defined limit of detection required for eradication campaigns.

## Discussion

Sensitive, low-cost and field-friendly methods of pathogen detection can significantly improve diagnosis of the neglected infectious diseases, and thus, the patient outcomes in resource-limited areas most severely affected by these diseases. This is especially true for malaria elimination campaigns in Africa. The current standard malaria diagnostics is based on microscopy and rapid diagnostic tools (RDT) that have the lower detection limit of approximately 50–100 parasites per µL of blood^14,15^, much higher than the WHO-recommended limit of ≤200 IE/100 µL. The modern highly sensitive methods that have the recommended detection limit, such as qPCR or LAMP, are expensive and hard to implement for wide-scale use in field studies^14^. The absence of a good field-deployable detection method is jeopardizing the recent achievements in global malaria control and the ambitious goal to eliminate or even eradicate malaria in the near future. There is an urgent need to develop a new diagnostic tool fulfilling the following criteria: high diagnostic accuracy for very low-density parasitemia cases, low cost, ease of use at point-of-care facilities in resource-limited settings, and potential for high-throughput design. Such diagnostics would provide the current malaria control programs and health care providers with a way to target curative treatment specifically to patients with confirmed infections. In turn, the precisely targeted treatment programs would minimize the risk of drug resistance development and spread, which is paramount considering the recent reports of emerging artemisinin resistance in SE-Asia^16^. The diagnostic tool would also assist in the ongoing and future malaria elimination efforts by detecting *P*. *falciparum* carriers with minute parasite densities, and thus, improve local, regional and global malaria surveillance. The current TORCA assay format still requires trained personnel, thus, further development would aim at further simplification and automation.

The TORCA method for nucleic acid detection described in this work can provide an attractive alternative to qPCR since it has similarly high sensitivity in the attomolar range achieved at a fraction of PCR-associated costs. The supreme specificity of various REase based DNA detection methods has been previously evaluated^6,7^, and has been proven for the TORCA assay in this study. The current TORCA assay time is <2 h, it is calculated as the sum of the recognition (2 × 40 min = 80 min) and amplification stages (15 min for bead mixtures) with several washing steps in-between. This recognition time is clearly excessive and can be shortened, especially for the low target concentration range. The amplification time can also be reduced by re-engineering of the amplification chamber to improve diffusion and minimize the bead-to-bead interactions in a way similar to our barrier-separated design. This can be done by using a barrier impermeable for the beads combined with a way to keep the beads suspended in solution, which may be achieved by alternating magnetic field, ultrasonic treatment, or mechanical agitation. Implementation of these measures can help to reduce the TORCA assay time to well below 1 h.

In addition to shortening of the assay time, the proposed re-engineering can greatly reduce backgrounds caused by bead-to-bead interactions. The background reduction can further improve the assay lower detection limit even below the currently achieved 10–100 attomolar concentrations. It will also improve reproducibility and reduce the need for multiple negative and positive controls to be analyzed alongside with patient samples. Further engineering will be associated with designing of a single microfluidics cartridge for all assay stages. Finally, the re-engineering efforts can help to further reduce the already low assay costs that are currently estimated to be between $1–2 per sample. By employing extra low volumes of assay components, the assay cost can be reduced to below $1.

Our data (Figs 6 and 7) demonstrate the proof-of-principle that TORCA can be used to detect malaria parasite infections in a wide range of parasitemia and at the concentrations close or below the WHO threshold (<200 IE/100 µL). The described experiments were done with low nucleic acid mass inputs: though the total RNA was extracted from 100 µL of blood, only one half of the resultant RNA mass was used for cDNA synthesis, and subsequently only 1/10 of the obtained cDNA mass was used for each individual TORCA assay reaction. Such low mass inputs were sufficient to reproducibly and robustly detect the 75 IE/100 µL parasite concentration, and to produce the above-background signals for the lowest concentration of 7.5 IE/100 µL. Thus, up to 20 different TORCA assays can be performed using the limited amount of material available from 100 µL blood. Alternatively, the TORCA assay performance can be improved by an order of magnitude by using all material obtained from the entire 100 µL blood sample for a single assay reaction. The volume of the recognition reaction can easily be altered to accommodate the concentrated sample mass. Such approach has great potential for detection of sub-microscopic infections at very low parasitemia levels.

The use of molecules highly expressed in *P*. *falciparum* parasites, like rRNA, as targets for detection provides for enhanced sensitivity. This study used the integrated signal obtained for the combined targets including the type A 18S rRNA and EEF2 mRNA. Further investigation of other potential RNA targets, and direct comparison of individual versus integrated assays is required to achieve the optimum performance of TORCA for the malaria detection.

Modern methods of RNA extraction are relatively simple for small size samples, amenable for high throughput designs, and adaptable for field use. The same is true for RNA conversion into cDNA using reverse transcription. The price for these manipulations makes the corresponding preparation steps affordable and field-friendly. The TORCA approach, after it is engineered and optimized as described above, could be further adapted for the high throughput field-friendly format, providing simple color outputs for digital quantification, for example, using smartphones. The molecular marker employed in the current assay is HRP, thus the signal detection can be done electrochemically, leading to the development of small battery-powered devices that can provide digital outputs. The future plans include optimization of the TORCA approach in terms of sensitivity, robustness and short assay time, and then TORCA application to a wide range of pathogens, including bacteria and viruses, to provide field-deployable and high throughput assays for resource-low health care settings around the world.

## Supplementary information

Supplementary Figure 1
