# Supplementary material for: Detection of sub-microscopic blood levels of Plasmodium falciparum using Tandem Oligonucleotide Repeat Cascade Amplification (TORCA) assay with an attomolar detection limit
Source: Sci Rep. 2019 Feb 27;9:2901. doi: 10.1038/s41598-019-39921-9 (PMC6393570; doi:10.1038/s41598-019-39921-9)

Detection of sub-microscopic blood levels of *Plasmodium falciparum* using Tandem Oligonucleotide Repeat Cascade Amplification (TORCA) assay with an attomolar detection limit

Andrey L. Ghindilis, Olga Chesnokov, Billy Ngasala, Maria W. Smith, Kenneth Smith, Andreas Mårtensson, and Andrew V. Oleinikov

Supplementary Information

Supplementary Figure 1. Calibration curve for RT-PCR quantification of parasitemia showing fluorescence of the DNA amplicons (Y-axis) corresponding to the simulated blood samples with different parasitemia (X-axis). Gel image quantification was done using Zen 2012 Blue software.

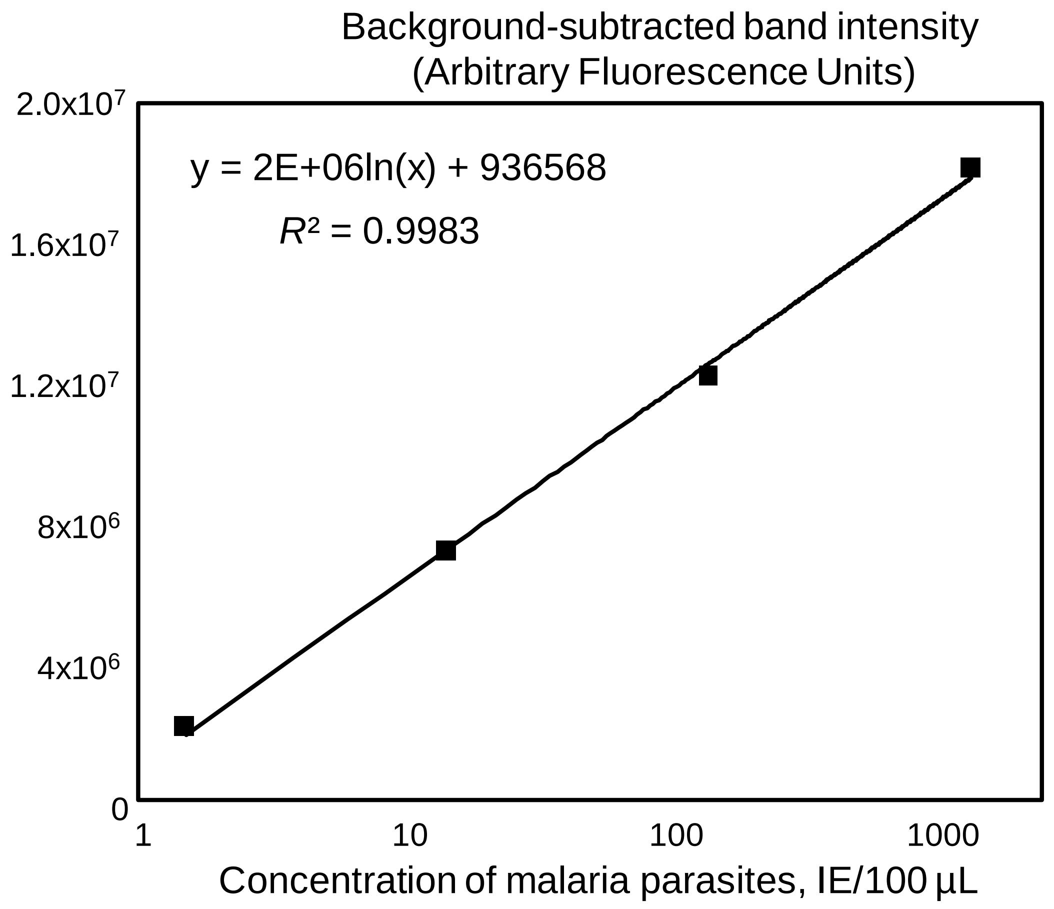

Supplement: Supplementary file 1 — Supplementary Figure 1 [file 41598_2019_39921_MOESM1_ESM.pdf]
